# Supplementary material for: Different Sensitivity of Advanced Bronchial and Alveolar Mono- and Coculture Models for Hazard Assessment of Nanomaterials
Source: Nanomaterials (Basel). 2023 Jan 19;13(3):407. doi: 10.3390/nano13030407 (PMC9921680; doi:10.3390/nano13030407)
Supplement: Supplementary file 1 [file nanomaterials-13-00407-s001.zip › nanomaterials-2089106-supplementary.pdf]

nanomaterials

MDPI

Supplementary materials

Different sensitivity of advanced bronchial and alveolar mono- and coculture models for hazard assessment of nanomaterials

Elisabeth Elje [1,2,*], Espen Mariussen [1,3], Erin McFadden [1], Maria Dusinska [1] and Elise Rundén-Pran [1,*]

[1] NILU—Norwegian Institute for Air Research, Department for Environmental Chemistry, Health Effects Laboratory, 2007 Kjeller, Norway
[2] University of Oslo, Institute of Basic Medical Sciences, Department of Molecular Medicine, 0372 Oslo, Norway
[3] Norwegian Institute of Public Health, FHI, 0456 Oslo, Norway
* Correspondence: eel@nilu.no (E.E.) ; erp@nilu.no (E.R.P.); Tel.: +47 63 89 81 98

S1. Cell cultures

The passage numbers for BEAS-2B, A549 and EA.hy926 cells used in this study is summarized in Table S1. The culture medium and supplements are summarized in Table S2.

Table S1. Passage numbers for BEAS-2B, A549 and EA.hy926 cells.

Culture	BEAS-2B	A549	EA.hy926
ALI monocultures	3,6,8,9,10,12,13,14	3,5,6,8,9,11,13,15	-
ALI cocultures	8,10,12,14	2,3,9,11,13,15	Coculture with BEAS-2B: 9,11,13,16.<br>Coculture with A549: 3,5,7,9,10.
Submerged monocultures	3,5,7,8,9,13	3,5,9,12	4,7,8,12,19

Table S2. Medium types used in mono- and cocultures, with supplements. DMEM: Dulbecco's modified Eagle's medium, with low glucose or high glucose; FBS: Fetal bovine serum; PS: penicillin-streptomycin. 1% PS equals 100 U/mL penicillin and 100 µg/mL streptomycin. % given as vol/vol.

Cell type	Medium	Serum	Supplement
**Monocultures**			
BEAS-2B	100 % LHC-9	-	-
A549	90 % DMEM low glucose	9 % FBS	1 % PS
EA.hy926	90 % DMEM high glucose	9 % FBS	1 % PS<br>4 mM L-glutamine<br>1 mM Sodium pyruvate
**Co-cultures**			
BEAS-2B/EA.hy926	50 % LHC-9<br>45 % DMEM high glucose	4.5 % FBS	0.5 % PS<br>2 mM L-glutamine<br>0.5 mM Sodium pyruvate
A549/EA.hy926	18 % DMEM low glucose<br>72 % DMEM high glucose	9 % FBS	1 % PS<br>3.5 mM L-glutamine<br>0.9 mM Sodium pyruvate

S1. Cytokinesis-block micronucleus assay

The semi-automatic scoring system identified mono- and binucleated cells. A minimum of 500 binucleated cells was scored for micronuclei of most samples, however, this was not possible for all samples due to low number of cells possible to score. The selected settings for scoring and analysis did not identify all binucleated cells with micronuclei, giving some false negative and false positive cells. Thus, all identified binucleated cells were manually accepted or rejected, and cells with possible micronuclei were checked with a 40x objective.

The percentage of binucleated cells in the populations used for micronucleus assay, was estimated by dividing the number of binucleated cells by the total number of analyzed cells. This is a minimum estimate, because the total numbers of analyzed cells were not checked in detail for all samples and thus may include some false positives/artefacts. Results are summarized in Figure S1. About 20% of the BEAS-2B and A549 cells were binucleated. EA.hy926 cells had about 19% binucleated cells when in coculture with BEAS-2B, and about 8% when in coculture with A549 cells.

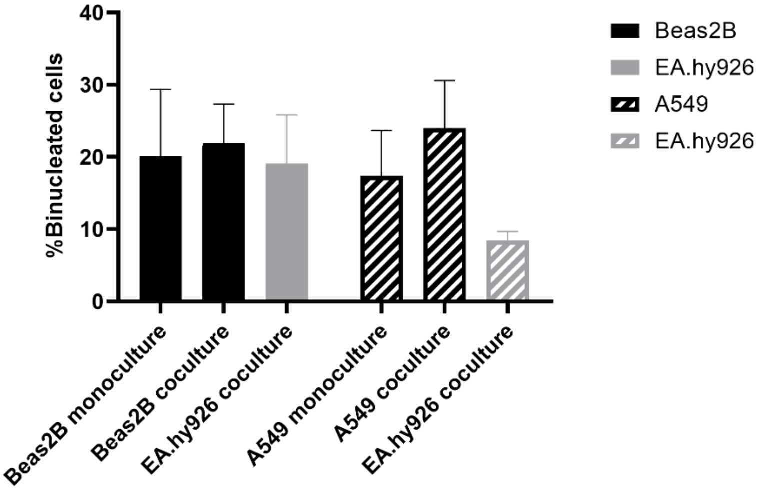

Figure S1. Percentage of binucleated cells in the samples for micronucleus assay. The numbers are minimum estimates.

S3. NM-300K concentrations for exposure

The concentrations of NM-300K applied for air-liquid interface exposure and submerged exposure are summarized in Table S3, with metrics mass per culture area and mass per volume of medium. The concentrations were corrected to measured Ag concentration in the stock dispersion by dividing the concentration by nominal stock concentration (10 mg/ml) and multiplying with corrected concentration (7.2 mg/ml).

Table S3. NM-300K concentrations applied for exposure, given as metrics mass per culture area and mass per volume of medium. ALI: air-liquid interface. *Corrected based on analysis of total Ag in the stock dispersion and on cell-free culture inserts, in our previous paper Camassa & Elje et al, 2022 [1].

	**Nominal concentrations**		**Corrected concentrations***	
	**μg/cm²**	**μg/ml**	**μg/cm²**	**μg/ml**
Submerged	0.1	0.1	0.1	0.1
	0.3	0.4	0.2	0.3
	1.0	1.4	0.7	1.0
	3.0	4.2	2.2	3.1
	10.0	14.1	7.2	10.2
	30.0	42.4	21.6	30.5
	75.0	106.0	54.0	76.3
	100.0	141.4	72.0	101.8
ALI low conc.	1	-	0.8	-
ALI high conc.	10	-	6.0	-

S4. Number of replica inserts and experiments

The number of cell culture inserts (= technical replica) per experiment for each test method is summarized in Table S4.

Table S4. Number of cell culture inserts per experiment. Models are described by cell types present; BEAS-2B (monoculture), BEAS-2B/EA.hy926 (coculture), A549 (monoculture), and A549/EA.hy926 (coculture). ELISA: enzyme-linked immunosorbent assay. MMC: mitomycin-C. CHLO: chlorpromazine hydrochloride.
* From our previous paper [1].

Test method	Model	Compartment	Exp	Number of inserts							Ref
				NC	PBS	NM-300K DIS	NM-300K low	NM-300K high	MMC	CHLO	
Fluorescein permeation	BEAS-2B	Basolateral	Exp1	2	2						
			Exp2	1	1						
	BEAS-2B/EA.hy926	Basolateral	Exp1	1	2						
			Exp2	1	1						
	Empty insert	Basolateral	Exp1	2							
			Exp2	2							
Ag permeation	BEAS-2B	Basolateral	Exp1			1	1	1			
			Exp2			1	1	1			
			Exp3				1	1			
	BEAS-2B/EA.hy926	Basolateral	Exp1			1	1	1			
			Exp2			1	1	1			
			Exp3				1	1			
	A549	Basolateral	Exp1			1	2	2			*
			Exp2			1		1			*
			Exp3				2	2			*
	A549/EA.hy926	Basolateral	Exp1			1		1			*
			Exp2			2	2	2			*
			Exp3				1				*
	Empty insert	Basolateral	Exp1					1			*
			Exp2					1			*
ELISA	BEAS-2B	Basolateral	Exp1	2	2	2	2	2			
			Exp2	2	2	1	2	2			
			Exp3	1	1	1	1	1			
			Exp4	1	1	1	1	0			
			Exp5	2	2	0	2	0			
			Exp6	2	0	0	0	0			
	BEAS-2B/EA.hy926	Basolateral	Exp1	2	1	1	1	1			
			Exp2	1	1	1	1	1			
			Exp3	2	1	1	1	1			

Table S4 continued.

ELISA	A549	Basolateral	Exp1	1	1	1	1	1		
			Exp2	2	2	2	2	2		
			Exp3	2	1	1	1	1		
	A549/EA.hy926	Basolateral	Exp1	2	2	2	2	2		
			Exp2	2	2	2	2	2		
alamarBlue	BEAS-2B	Apical	Exp1	2	2					2
			Exp2	2	2		2			1
			Exp3	2	2	2	2	2	2	1
			Exp4	2	2	1	2	2		
			Exp5	2	1	1	1	1	1	1
			Exp6	1	1	1	1	1	1	1
			Exp7	2	1	1	1	1	1	1
	BEAS-2B/EA.hy926	Apical	Exp1	2	1	1	1	1	1	1
			Exp2	1	1	1	1	1	1	1
			Exp3	2	1	1	1	1	2	1
			Exp4	2	1	1	1	1	0	1
		Basolateral	Exp1	2	1	1	1	1	1	1
			Exp2	1	1	1	1	1	1	1
			Exp3	2	1	1	1	1	2	1
			Exp4	2	1	1	1	1	0	1
Comet assay	BEAS-2B	Apical	Exp1	2	2	2	2	2		
			Exp2	2	2	1	2	2		
			Exp3	1	1	1	1	1		
			Exp4	1	1	1	1	0		
			Exp5	2	2	0	2	0		
			Exp6	2	0	0	0	0		
	BEAS-2B/EA.hy926	Apical	Exp1	2	1	1	1	1		
			Exp2	1	1	1	1	1		
			Exp3	2	1	1	1	1		
		Basolateral	Exp1	2	1	1	1	1		
			Exp2	1	1	1	1	1		
			Exp3	2	1	1	1	1		
			Exp4	2	1	1	1	1		

Table S4 continued.

Micronucleus	BEAS-2B	Apical	Exp1	2	1	1	1	1	1
			Exp2	1	1	1	1	1	1
			Exp3	2	1	1	1	1	1
	BEAS-2B/EA.hy926	Apical	Exp1	2	1	1	1	1	1
			Exp2	1	1	1	1	1	1
			Exp3	2	1	1	1	1	1
		Basolateral	Exp1	2	1	1		1	1
			Exp2	1	1	1	1	1	1
			Exp3	2	1	1	1	1	1
	A549	Apical	Exp1	1		1	1	1	1
			Exp2	2	2	2	2	2	2
			Exp3	2	1	1	1	1	1
	A549/EA.hy926	Apical	Exp1	2	2	2	2	2	1
			Exp2	2	2	2	2	2	2
			Exp3	2	2	2	2	2	2
		Basolateral	Exp1	2	2	2	2	2	2
			Exp2	2	2	2	2	2	2
			Exp3	2	2	2	2	2	2

S5. Advanced models

The advanced models of epithelial and endothelial cells cultured on permeable inserts were moist with a shiny surface when cultured in ALI. However, occasionally a lack of moisture/shine was seen (Figure S2). The parts with more dry appearance were located in variable positions on the insert. This was occasionally seen in all types of cultures and was not systematically investigated. Confocal microscopy of the BEAS-2B/EA.hy926 co-cultures, indicated that the BEAS-2B cells were growing in multilayers (Figure S3).

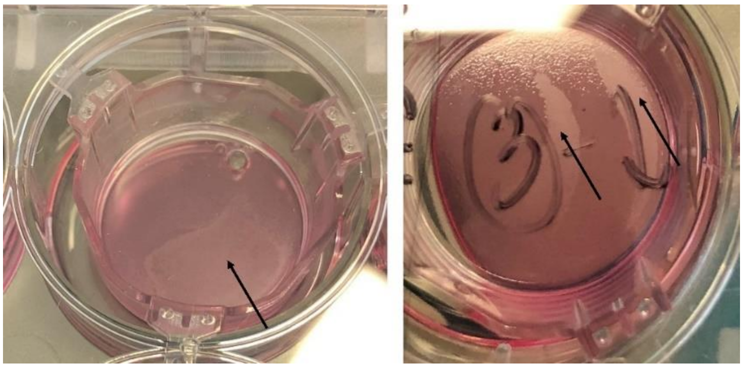

Figure S2. Images of A549/EA.hy926 cocultures at the ALI, cultured on permeable 1 µm 6 well inserts. Occasionally, a lack of moisture/shine was seen, marked by black arrows in the images.

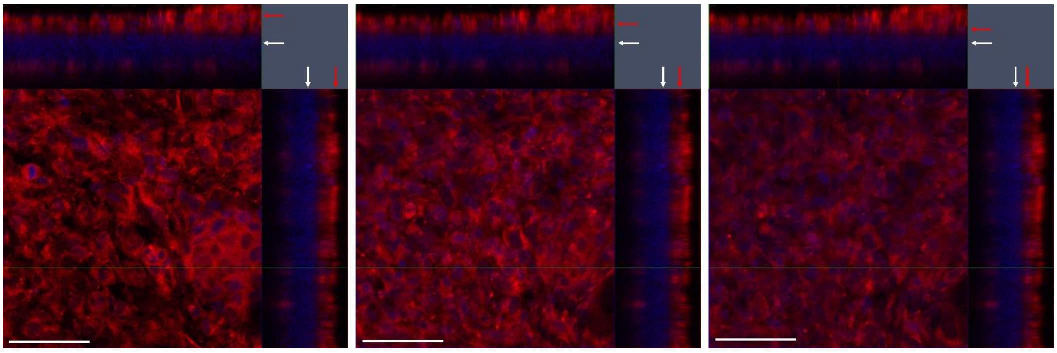

Figure S3. Confocal images of advanced bronchial models. Z-stack image series (2D x-y view and respective side views) showing the distribution of BEAS-2B on the apical side sides of a transwell insert (white arrows), at different heights from the insert (red arrows). The distance from the insert is decreasing from left to right. Z-stack thickness 52 µm. Red: cellular membranes stained with Cell Mask red dye, blue: nuclei counterstained with DAPI. Magnification: 40x. Scale bars 50 µm.

S6. AlamarBlue assay on submerged cells after NM-300K exposure

For comparison of the new advanced models with corresponding traditional cell models, the cytotoxicity of NM-300K was tested also with submerged exposure of mono-cultured cells by alamarBlue assay. BEAS-2B cells (P5-13) were seeded at a density of 15.000 cells/well ($5.3x10^4$ cells/cm^2) in 96-well plates pre-coated with Collagen IV. Pre-coating was performed for BEAS-2B only, with 40 µl of 30 µg/ml Collagen IV overnight at 4 °C, and plates were washed with PBS and used immediately. A549 (P3-12) were seeded in 96-well plates at 10.000 cells/well (3.5 $x10^4$ cells/cm^2), and EA.hy926 (P4-19) at 10.000 cells/well (3.5 $x10^4$ cells/cm^2) or 15.000 cells/well ($5.3x10^4$ cells/cm^2). Equal cell density was used for all samples within each experiment. The cells were incubated overnight at 37 °C and 5 % CO_2. The next day, the cells were exposed to nominal concentrations 0.1-100 µg/cm^2 NM-300K diluted in culture medium (200 µl/well) for 24 h. For corresponding mass/volume and corrected concentrations, see Table S3. BEAS-2B cells were also exposed to corresponding concentrations of NM-300K DIS (solvent control), diluted in cell culture medium. Negative control was exposed to culture medium only. Positive control chlorpromazine hydrochloride was prepared as described in main article and exposed to the cells in the apical media (200 µl/well) for 24 h. Cells were exposed in duplicates or triplicates.

AlamarBlue assay was performed on submerged cultures as described in section 2.11 in the main article, except with smaller volumes and longer incubation time. After exposure, the cells were washed twice with 100 µl PBS before 200 µl alamarBlue solution (10 % v/v) was added to each well. Aliquots were taken in triplicates (40 µl/well) for fluorescence reading after 3 h incubation at 37 °C and 5 % CO_2. Negative control was cells exposed to culture media only.

The viability of BEAS-2B, A549 and EA.hy926 cells, relative to cells exposed to culture medium only (set to 100 %), was affected by NM-300K (Figure S4 and Table S5) and NM-300K DIS (Figure S4). BEAS-2B cells were more sensitive to NM-300K exposure compared to A549 and EA.hy926 cells. The effective concentration giving 50 % cytotoxicity (EC_{50}) was 16.4 µg/cm^2 for BEAS-2B and about the double for A549 (Table S5, [2, 3]). The positive control chlorpromazine hydrochloride reduced the viability as expected (by >80 %, not shown). NM-300K DIS was cytotoxic at concentrations equivalent to 37 µg/cm^2 NM-300K, and no effect was seen at the concentration equivalent to about 10 µg/cm^2 (Figure S4).

To test if the NM-300K interfered with the readout of the AB assay, separate cell-free samples with NM-300K and AB solution were analyzed. NMs were diluted in LHC-9 culture medium to concentrations 0.1-100 µg/cm^2 and mixed with 10% (v/v) AB solution,

including blank sample (AB-medium without NMs), to a final volume of 200 µl per well in a 96 well plate. The plates were incubated at 37 °C and 5 % CO_2 for approximately 3h. The solution was mixed by pipet, and 40 µl aliquots were transferred in duplicate to black 96 well plates, before fluorescence (excitation 530 nm, emission 590 nm) was measured on a FLUOstar OPTIMA microplate reader. Results were averaged and compared to blank. Three independent experiments were performed, each with 1 well per concentration and at least 2 blanks. No difference was seen in fluorescence intensity from samples with NMs compared to blank, which indicate no interference between the NM-300K and the AB assay (Figure S5).

Table S5. Calculated EC_{50} (effective concentration giving 50 % cytotoxicity) values for cytotoxic effect of NM-300K on submerged monocultures of BEAS-2B, A549 and EA.hy926 cells measured by the alamarBlue assay after 24h exposure. Values are given with dose metrics mass per surface (µg/cm²) and mass per volume (µg/ml) ± standard deviation, based on nominal concentrations. Results are calculated from at least 2 independent experiments (n=2) and presented with standard deviation (BEAS-2B, EA.hy926) or standard error of the mean (A549).

Cell line	EC_{50} µg/cm²	EC_{50} µg/ml	Reference
BEAS-2B	16.4 ± 6.0	23.2 ± 8.4	-
A549	37 ± 15	59 ± 43	[2]
EA.hy926	22.5 ± 8.2	36.0 ± 13.1	[3]

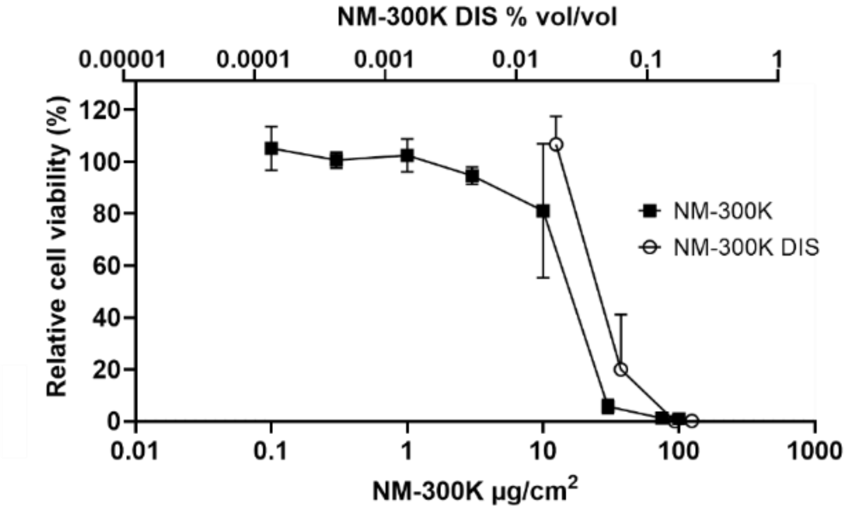

Figure S4. Relative cell viability of BEAS-2B cells after 24h submerged exposure to NM-300K (black squares) and NM-300K DIS (open circles), evaluated by alamarBlue assay. Results are presented as mean with SD from duplicate culture wells from n=4 independent experiments. Positive control (chlorpromazine hydrochloride 50-100 µM for 24h) gave a relative viability of <20 % (not shown in figure). Statistically significant different effects on cell viability compared to negative control cultures with cells exposed to medium (0 µg/cm²) were analyzed by one-way ANOVA followed by Dunnett´s post-hoc test (*P<0.05, **P<0.01, *** P<0.001). SD: standard deviation.

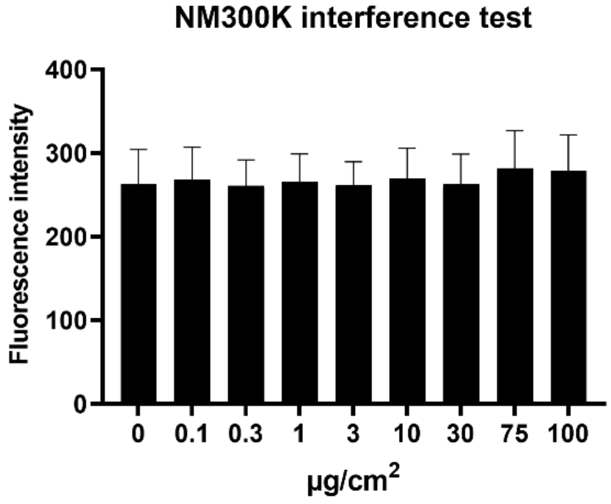

Figure S5. Alamarblue interference test, n=3 with single wells. No statistically significant different from blank (0 µg/cm^2), tested by one-way ANOVA followed by Dunnett´s post-hoc test. Nominal concentrations are shown. Please see Table S1 for concentrations as mass/volume and corrected after ICP-MS analysis.

S7. Enzyme-linked immunosorbent assay (ELISA)

The concentrations of IL-6 and IL-8 measured by ELISA are summarized in Table S6 and S7, and relative concentrations (relative to NC) in Figure S6. Potential interference between NM-300K and the performance of the ELISA was investigated. NM-300K was prepared as described in the main document, and diluted in cell culture media for BEAS-2B cells, A549 cells, or EA.hy926 cells, to achieve the concentrations 30, 3 and 0.3 µg/ml. The NMs in media were added to the ELISA plate in duplicates and mixed with reagent buffer or standard (25 pg/ml IL-6 or 31 pg/ml IL-8) provided in the kit. Further steps in the assay were run as described for the other samples (in main document). The results showed no interference between the NM-300K and the assay. The culture medium with and without NM-300K had similar absorbance as the blank samples of the kit. The combination of IL-6 or IL-8 and NM-300K had similar measured interleukin concentration as the NM-free control samples (Figure S7).

Table S6. Concentrations of interleukin 6 (IL-6) by the enzyme-linked immunosorbent assay (ELISA). Mean ± standard deviation (n independent experiments).

Treatment	BEAS-2B	BEAS-2B/EA.hy926	A549	A549/EA.hy926
NC	304 ± 158 (n=6)	875 ± 203 (n=3)	13 ± 13 (n=3)	8 ± 2 (n=2)
PBS	1229 ± 313 (n=6)	1758 ± 364 (n=3)	23 ± 7 (n=2)	13 ± 4 (n=2)
NM-300K_DIS	1744 ± 461 (n=5)	2604 ± 138 (n=3)	15 ± 6 (n=3)	11 ± 2 (n=2)
NM-300K_low	1647 ± 432 (n=6)	4595 ± 2842 (n=3)	19 ± 9 (n=3)	11 ± 1 (n=2)
NM-300K_high	542 ± 189 (n=5)	2501 ± 910 (n=3)	26 ± 10 (n=3)	13 ± 2 (n=2)

Table S7. Concentrations of interleukin 8 (IL-8) by the enzyme-linked immunosorbent assay (ELISA). Mean ± standard deviation (n independent experiments).

Treatment	BEAS-2B	BEAS-2B/EA.hy926	A549	A549/EA.hy926
NC	660 ± 444 (n=6)	5991 ± 1838 (n=3)	2695 ± 1637 (n=3)	725 ± 238 (n=2)
PBS	1640 ± 921 (n=6)	9140 ± 2135 (n=3)	2366 ± 579 (n=2)	1377 ± 27 (n=2)
NM-300K_DIS	2354 ± 1693 (n=5)	10728 ± 2541 (n=3)	2996 ± 1416 (n=3)	1534 ± 525 (n=2)
NM-300K_low	3500 ± 2903 (n=5)	22672 ± 16736 (n=3)	3580 ± 1830 (n=3)	1227 ± 545 (n=2)
NM-300K_high	1618 ± 692 (n=5)	14053 ± 1308 (n=3)	3969 ± 2360 (n=3)	1816 ± 246 (n=2)

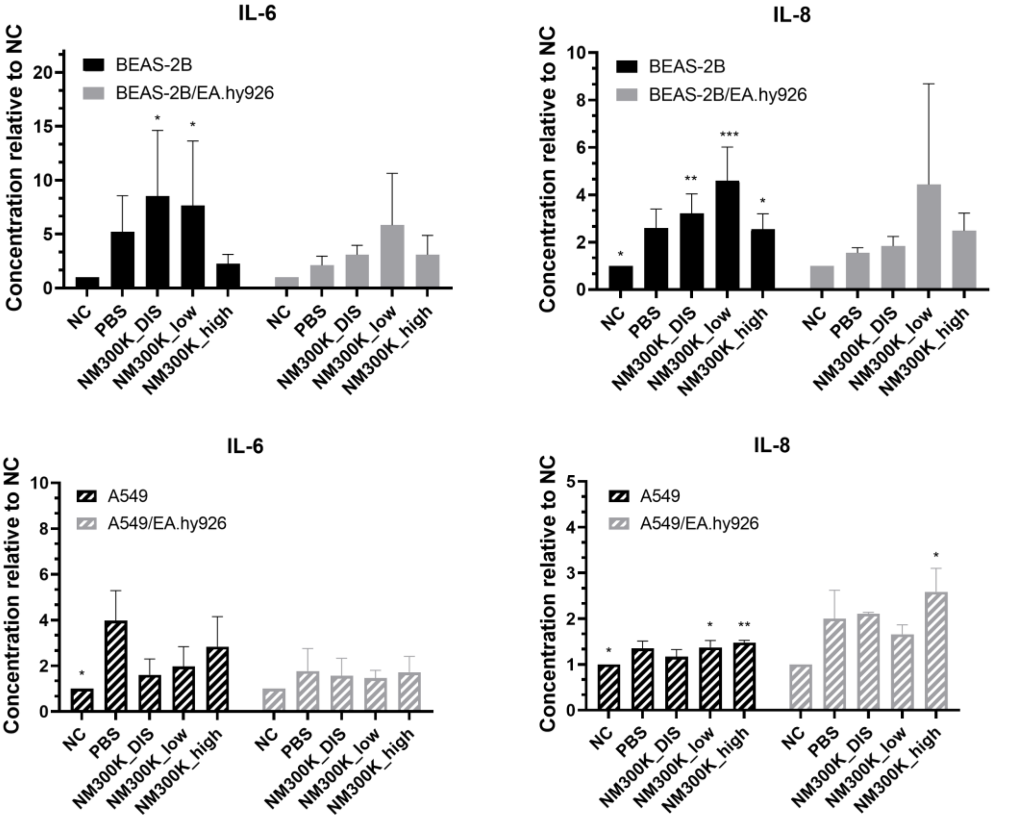

Figure S6. Relative concentrations of IL-6 and IL-8 in mono- and co-cultures of BEAS-2B/EA.hy926 cells (top panel) and A549/EA.hy926 cells (bottom panel) after exposure to aerosolized NM-300K and control solutions at the air-liquid interface, evaluated by ELISA. Results are presented as mean with SD from single or duplicate inserts from n=2-6 independent experiments (n=6 for BEAS-2B monocultures, n=3 for BEAS-2B/EA.hy926 cocultures and A549 monocultures, and n=2 for A549/EA.hy926 cocultures). Statistically significant different effects on cell viability compared to negative control inserts with non-exposed cells (NC) were analyzed by one-way ANOVA followed by Dunnett´s post-hoc test (*P<0.05, **P<0.01, *** P<0.001). NC: negative control; PBS: Phosphate buffered saline; SD: standard deviation.

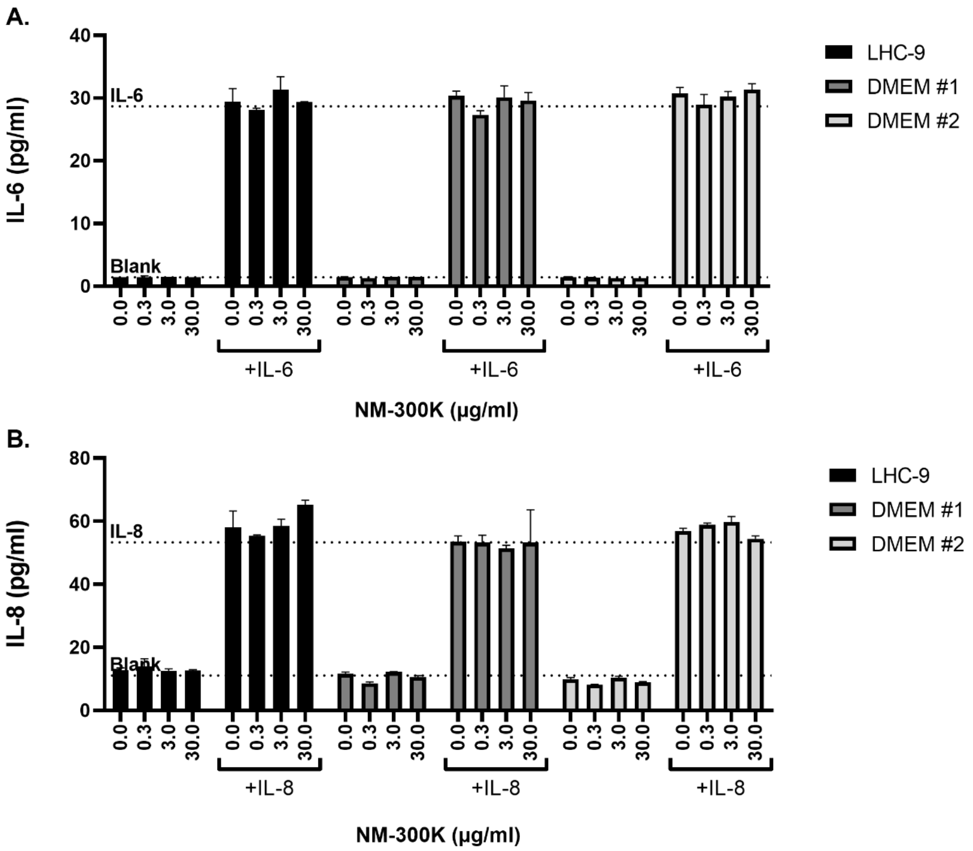

Figure S7. ELISA interference test of NM-300K in cell culture media. No interference of NM-300K with the ELISA was found, as the measured concentrations of interleukin 6 (A) and 8 (B) in samples with mixture of NM-300K, cell culture medium, and interleukins were in the same range as the corresponding controls. The test was run with duplicate samples. LHC-9 (black): medium for BEAS-2B cells, DMEM #1 (dark grey): medium for A549 cells, DMEM #2 (light grey): medium for EA.hy926 cells.

S7. Comet assay on submerged cells after NM-300K exposure

The induction of DNA damage after exposure to NM-300K was measured by the comet assay. Cells were exposed as described above for alamarBlue assay on submerged cultures. Comet assay was performed as described in section 2.13 in the main article. First, cells were washed twice with 100 µl PBS, and detached by dry trypsinization for 3-5 min at 37 °C and 5 % CO_2. Cells were resuspended in 150 µl medium. Immediately after, 50 µl cell suspension was mixed with 200 µl LMP-agarose. Separate cell cultures were used for alamarBlue and comet assays. Potential interference between NM-300K and the readout of the comet assay was investigated as described in [39].

NM-300K caused increased levels of DNA SBs and oxidized base lesions at cytotoxic concentrations only (Figure S8). A slight increase in SBs was seen at 10 µg/cm^2. However, no effect was seen at 1 µg/cm^2, corresponding to the concentration giving DNA damage after ALI exposure of BEAS-2B monocultures. No interference was seen (not shown).

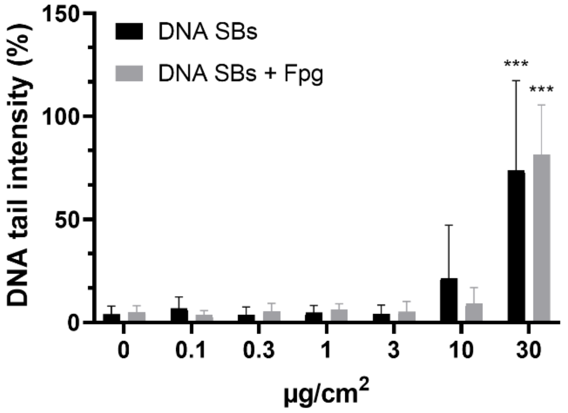

Figure S8. DNA damage by strand breaks (SBs) and oxidized base lesions (SBs + Fpg) in BEAS-2B cells after 24 hours submerged exposure to NM-300K (nominal concentrations) evaluated by Fpg-modified comet assay. Results are presented as mean with SD from n=3 independent experiments, each with duplicate exposure wells, except for DNA SBs + Fpg 10 and 30 µg/cm^2 where n=2. Statistically significant different effects on DNA damage compared to negative control cultures with cells exposed to medium (0 µg/cm^2) were analyzed by one-way ANOVA followed by Dunnett´s post-hoc test (*p<0.05, **p<0.01, ***p<0.001). SBs: strand breaks; SD: standard deviation.

S7. Effect of medium composition on cyto- and genotoxicity in BEAS-2B and EA.hy926 cells

Different media compositions were used in the BEAS-2B monocultures (LHC-9) and in the BEAS-2B/EA.hy926 cocultures (DMEM and LHC-9, 1:1), and we wanted to investigate if this could influence on the different sensitivity in the measured cyto- and genotoxicity of the cells. Thus, the relative viability and DNA damage responses of submerged BEAS-2B or EA.hy926 cells were evaluated after 24 h exposure to LHC-9 and DMEM media at different ratios (10-100 %).

No difference in relative viability (Figure S9) or background levels of DNA SBs or Fpg sites (not shown) was seen on either of the cell types with the different media compositions, compared to cells cultured in the cells' respective media. Induction of DNA damage by H_2O_2 exposure in the gels was similar for the submerged cells with different media compositions (Figure S10). The submerged BEAS-2B cells had less sensitivity to H_2O_2 compared to BEAS-2B cells from mono- or cocultures at ALI conditions (Table 5 in article). Rather high background of DNA SBs was seen in EA.hy926 cells, and less sensitivity to H_2O_2 compared to BEAS-2B cells, independently on the culture media (Figure S10).

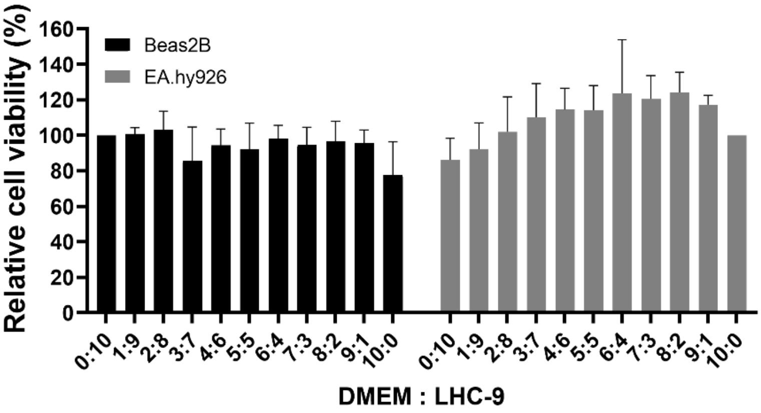

Figure S9. Relative cell viability of BEAS-2B cells after 24h submerged exposure to DMEM and LHC-9 culture media, evaluated by AlamarBlue assay. Results are presented as mean with SD from triplicate culture wells from n=3 independent experiments. Positive control (chlorpromazine hydrochloride 100 µM for 24h) gave a relative viability of <5 % (not shown in figure). Statistically significant different effects on cell viability compared to negative control cultures with cells exposed to the respective culture medium (LHC-9 for BEAS-2B, DMEM for EA.hy926) were analyzed by one-way ANOVA followed by Dunnett´s post-hoc test. No statistically significant results were found (P>0.05). SD: standard deviation.

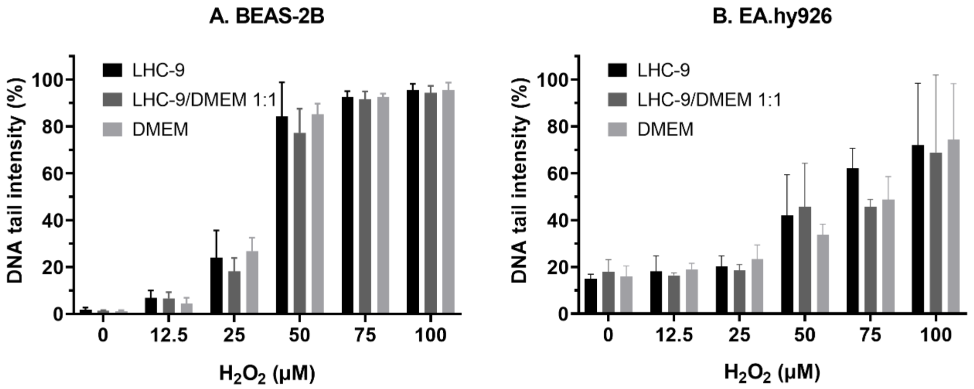

Figure S10. DNA strand breaks (SBs) evaluated by the comet assay after exposure to hydrogen peroxide (H_2O_2) of monocultures of BEAS-2B (A) and EA.hy926 (B) cells cultivated at submerged conditions with different media compositions. Results are presented as mean with SD from duplicate culture wells from n=3 independent experiments. SBs: strand breaks, SD: standard deviation.

References

1. Camassa, L. M. A.; Elje, E.; Mariussen, E.; Longhin, E. M.; Dusinska, M.; Zienoldddiny-Narui, S.; Rundén-Pran, E. Advanced Respiratory Models for Hazard Assessment of Nanomaterials—Performance of Mono-, Co- and Tricultures. Nanomaterials 2022, Vol. 12, Page 2609 2022, 12 (15), 2609. https://doi.org/10.3390/NANO12152609.

2. El Yamani, N.; Mariussen, E.; Gromelski, M.; Wyrzykowska, E.; Grabarek, D.; Puzyn, T.; Tanasescu, S.; Dusin-ska, M.; Rundén-Pran, E. Hazard Identification of Nanomaterials: In Silico Unraveling of Descriptors for Cytotoxicity and Genotoxicity. Nano Today 2022, 46, 101581. https://doi.org/10.1016/J.NANTOD.2022.101581.

3. Rundén-Pran, E. Manuscript in Preparation. 2023.
